# The serum levels of triacylglycerols, nonesterified fatty acids, and beta-hydroxybutyrate as markers of reproductive capability of primiparous dairy cows

**DOI:** 10.1093/jas/skaf276

**Published:** 2025-09-05

**Authors:** Tereza Toralová, Jaromír Ducháček, Matúš Gašparík, Veronika Kinterová, Nikola Marešová, Radim Codl, Luděk Stádník

**Affiliations:** Laboratory of Developmental Biology, Institute of Animal Physiology and Genetics CAS, Liběchov, Czech Republic; Department of Animal Science, Faculty of Agrobiology, Food and Natural Resources, Czech University of Life Sciences Prague, Prague, Czech Republic; Department of Animal Science, Faculty of Agrobiology, Food and Natural Resources, Czech University of Life Sciences Prague, Prague, Czech Republic; Laboratory of Developmental Biology, Institute of Animal Physiology and Genetics CAS, Liběchov, Czech Republic; Department of Animal Science, Faculty of Agrobiology, Food and Natural Resources, Czech University of Life Sciences Prague, Prague, Czech Republic; Department of Animal Science, Faculty of Agrobiology, Food and Natural Resources, Czech University of Life Sciences Prague, Prague, Czech Republic; Department of Animal Science, Faculty of Agrobiology, Food and Natural Resources, Czech University of Life Sciences Prague, Prague, Czech Republic

**Keywords:** cumulus cells, metabolic stress, negative energy balance, oocyte aspiration, oocyte quality, primiparous dairy cows

## Abstract

Metabolic stress and negative energy balance (**NEB**) are typical undesirable accompanying phenomena of the postpartum period in dairy cattle. They negatively affect not only milk production but also the reproductive abilities of the cow, and it is therefore desirable to recognize NEB early to prevent its development. Metabolic stress markers are traditionally total cholesterol (tChol), nonesterified fatty acids (**NEFA**), beta-hydroxybutyrate (BHB), and triacylglycerols (TAGs). The aim of this work was to determine whether the level of the aforementioned markers in the blood correlates with the ability of a primiparous dairy cow to conceive soon after the first calving. Therefore, oocytes were collected from the monitored cows shortly after calving using the ovum pick-up method, and their quality was subsequently determined. We observed cumulus cell expansion, oocytes quality, maturation rate, and the amount and distribution of lipids within the oocyte. In addition to the mentioned markers, we also monitored the effect of the season in which aspiration was performed to assess its impact on the reproductive indicators of the monitored cows. We observed a negative correlation between higher TAGs and oocyte maturation rate, while the number of aspirated oocytes per cow and the fertilization capability were positively associated with TAGs. Additionally, higher BHB levels in the blood were linked to enhanced fertilization capability. The higher levels of TAGs, BHB, and NEFA in blood were associated with increased lipid intensity, and higher lipid content was positively correlated with both the quality of cumulus cells and oocytes. In summary, we found that NEFA, BHB, and TAGs are the most reliable markers of a cow’s readiness to become pregnant after her first calving. The use of tChol, however, remains very controversial. Moreover, primiparous cows are more prepared for re-pregnancy in the warmer season.

## Teaser

This study describes markers of metabolic stress that are the most promising candidates for identifying cows that display early postpartum reproductive capacity.

## Graphical Abstract

**Graphical Abstract:**
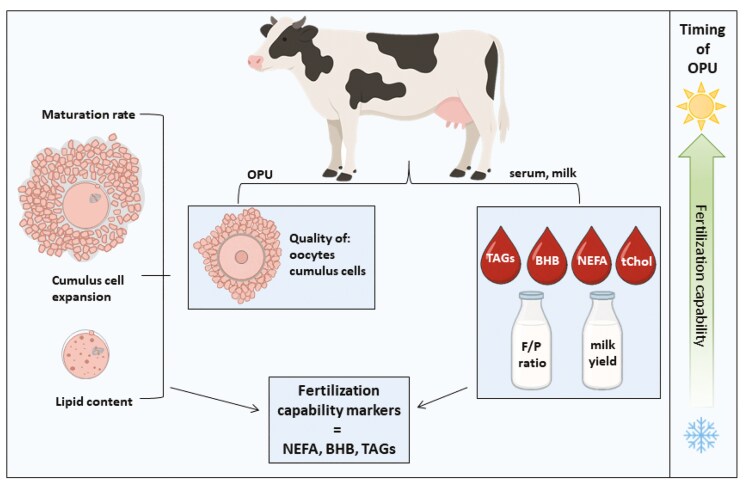

## Introduction

Metabolic stress is associated with the postpartum period and a state of negative energy balance (**NEB**), which is associated with endocrine and metabolic consequences on the organism (Leroy et al., 2008a). The plasma concentrations of triacylglycerols (TAG), beta-hydroxybutyrate (BHB), and nonesterified fatty acids (**NEFA**) increase within NEB period, but the concentrations of cholesterol and glucose levels decrease at the same time (Kawashima et al., 2007; Song et al., 2021). However, other studies have recorded an increase in cholesterol levels after calving (Leroy et al., 2004; Takahashi et al., 2021). This discrepancy may be related to the fact that multiparous cows tend to have higher cholesterol levels compared to primiparous cows (Yehia et al., 2020). The postpartum period of mammals is characterized by enhanced lipid mobilization, which results in altered lipid content in serum (Tessari et al., 2020), fatty acids in milk (Stádník et al., 2015), and various metabolites in cervical mucus such as urea or acetone (Beran et al., 2013). The serum concentration of BHB, NEFA, and other metabolites can be used to monitoring subclinical ketosis and therefore NEB (Leroy et al., 2008b; Tufarelli et al., 2024). NEB, change in milk composition or the ratio of fat to protein (Stádník et al., 2022), and the increased concentration of metabolites in this period have a demonstrable effect on the deterioration of the health and reproduction of dairy cows (Bezdíček et al., 2024). According to Britt (2008), follicles and oocytes undergoing early development are at risk during NEB. According to Leroy et al. (2013), the disturbed metabolism of the dairy cow can change the endocrine and biochemical composition of the follicular fluid and thus the microenvironment of the oocyte.

The oocyte maturation, its quality, and ability to produce a viable embryo is a key factor of reproduction and is affected by several different factors. In vivo maturation responses mainly to the health status of the cow. In vitro maturation success is largely influenced by the maturation media and culture conditions. The maturation environment can thus affect not only the maturation of the oocyte but also its gene expression (Bartková et al., 2024). The oocyte maturation is a gradual process consisting of many substeps. In case of in vitro culture of oocytes and embryos of farm animals, the germinal vesicle (**GV**; germinal vesicle—the nucleus of oocyte) oocytes are in vitro matured into fertilizable metaphase II (MII) oocytes. While oocytes at GV stage are arrested at prophase of the first meiosis and are still not fully matured, the MII oocytes are arrested at metaphase II of the second meiotic division and are prepared to be fertilized. After fertilization, the resumption and completion of meiosis occur and fluently continues to the embryonic development. For the proper maturation, not only the quality of the oocytes is needed, but also the quality of the surrounding cumulus cells and their ability to expand. The production of extracellular matrix and consequent expansion of cumulus cells enables the proper maturation of the oocyte. The quality of cumulus cells is a key factor needed for proper communication and signal transfer between them and the oocyte (Martinez et al., 2023).

The productivity of dairy cows has been continuously evolving due to intensive breeding, which is also reflected in physiological changes and stress responses at the organismal level. The main contribution of this study therefore lies in monitoring the current status and trends in the reproductive performance of Holstein cattle in the Czech Republic. We focus on newly obtained data that reflect the ongoing influence of genetic selection and breeding on the fertility and metabolism of the cow. The hypothesis of this work was that cows in NEB may produce different concentrations of metabolites, which influence the quantity and quality of aspirated oocytes. The aim of this study is to describe relationships between tChol, TAGs, NEFA, BHB concentration, and quality of aspirated oocytes.

## Materials and Methods

### Animals

This experiment was carried out in accordance with the Czech legislation for Protection of Animals against Cruelty (Act No. 246/1992) and with Directive 2010/63/EU on Protection of Animals Used for Scientific Purposes. The animals in the experiment were housed on a commercial dairy farm in the Central Bohemian Region of the Czech Republic. All tested cows were Holstein breed. Only cows (*n* = 92) at their first lactation and diagnosed as cycling (corpus luteum present on the ovary) were selected and then aspirated. Cows were milked twice daily, and the composition of the total mixed ration was adjusted according to the daily milk yield of this group of dairy cows.

### Oocyte retrieval—ovum pick-up

All cows in the experiment were at early lactation. The first ultrasound-guided follicular aspiration in donor cows was done on average at 58.05 d in milk (**DIM**) with a minimum of 36 DIM and a maximum of 75 DIM. Usually, a second aspiration followed a week later if no problems were detected. In total, 155 successful aspirations were performed between 2020 and 2023. Cows were treated with epidural anesthesia (4 mL of 2% Lidocaine, Fatro, Italy), and afterwards, the rectum and vulva were cleaned. The ovaries were visualized with a 7 MHz convex probe attached to an ultrasound scanner MyLabOne VET (Esaote, Genova, Italy). An 18 G disposable hypodermic needle was fitted to a custom-made intravaginal probe holder, and—75 mm Hg pressure was applied using a vacuum aspiration pump. A 50 mL tube for the retrieval of cumulus-oocyte complexes (**COCs**) was held at 37 °C and filled with commercial oocyte retrieval media (OPU, Cornwall, UK). Immediately after each aspiration, the content of the 50 ml tube was filtered through an embryo filter, and COCs were searched under a stereomicroscope. Retrieved oocytes were transferred in a commercial holding medium (BO-IVM, Cornwall, UK) to the laboratory within 2 h after ovum pick-up (**OPU**). This methodology is described in Stádník et al. (2024).

### Blood samples

A total of 184 blood samples were collected from 92 cows with always two rounds of aspirations. The blood samples were collected from the *coccygeal vein* using 1.20 × 25 mm single-use needles into 4 mL BD Vacutainer rapid serum tubes (Becton Dickinson, Franklin Lakes, NJ, USA) before oocytes aspiration. After collection, the blood was allowed to clot by leaving it undisturbed at room temperature (approximately 22 °C) for 24 h (Menéndez et al., 2001). Thereafter, the sera were transferred into 1.5 mL Eppendorf Safe Lock microtubes (Eppendorf, Hamburg, Germany) using Pasteur pipettes. Serum samples were stored at −18 °C until biochemical analysis. The biochemical analysis was performed at the Large Animal Clinical Laboratory of the University of Veterinary Science Brno, Brno, Czech Republic, within a maximum of 3 mo after sample collection. Sera was analyzed for total cholesterol (tChol; mmol/l), TAGs (mmol/l), NEFA (free fatty acids; mmol/L), and BHB (mmol/L) concentrations using the enzymatic endpoint method, using DiaSys commercial diagnostic kits (Diagnostic Systems, Holzheim, Germany) and a Konelab 20XT automatic biochemical analyzer (Thermo Fisher Scientific, Waltham, MA, USA). This methodology is described in Štolcová et al. (2024).

### Milk yield and fat/protein ratio

Daily milk yield (liters of milk), fat, and protein content were recorded by Afifarm (v4.1, Afikim, Israel). The fat/protein ratio was subsequently calculated. The chosen periods of data acquisition were as follows: gathered within a period of 25 to 35 DIM, at the time of OPU and in the period from calving to OPU.

### In vitro maturation

Unless otherwise indicated, the chemicals were purchased from Sigma (Sigma-Aldrich, St. Louis, MO) and plastic from Nunclon (Nunc, Roskilde, Denmark). In vitro maturation was performed as stated in Benešová et al. (2017).

After arrival of COCs to laboratory, these were thoroughly washed and subjected to in vitro maturation in Modified Parker medium supplemented with 20 mM sodium pyruvate, 50 U/mL penicillin, 50 µg/mL streptomycin, 10% fetal bovine serum, serum gonadotropin, and chorionic gonadotropin (P. G. 600, 15 U/mL; Intervet, Boxmeer, Holland) without a paraffin overlay in four-well dishes under a humidified atmosphere for 24 h at 39 °C with 5% CO2. After 24 h, oocytes were denuded by gentle pipetting and fixed.

To determine cumulus cells expansion and evaluate quality, COCs or denuded oocytes were scanned by Axiocam 208 color (Zeiss, Oberkochen, Germany) before and after maturation.

The oocytes were classified as capable when at least one of the oocytes aspirated from one cow reached the MII stage.

**Figure 1.**
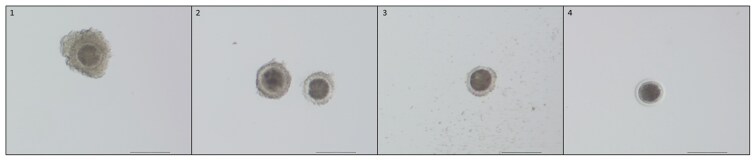
Representative images of COCs classification. Classes: (1) more than five layers of CCs; (2) 2 to 5 layers; (3) one layer (also partly surrounding); (4) no cumulus cells. Scale bars indicate 200 µm.

**Figure 2.**
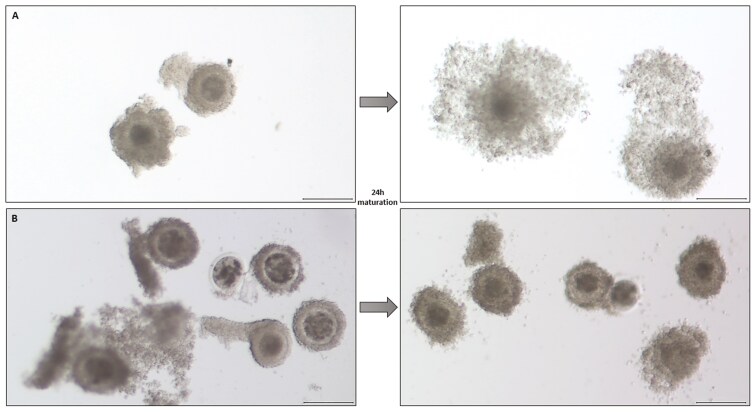
Representative images of cumulus cell expansion. COCs before and after 24 h of maturation with (A) successfully expanded cumulus and (B) without cumulus expansion. Scale bars indicate 200 µm.

**Figure 3.**
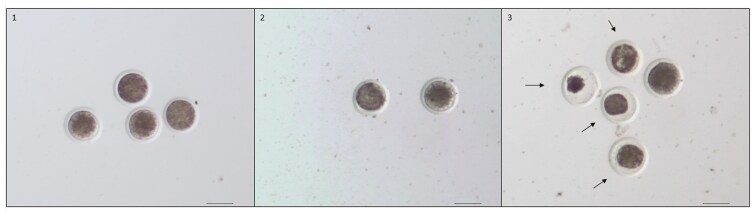
Representative images of oocytes classification. Classes: (1) Compact, homogeneous; (2) granulated, heterogeneous; (3) fragmented or heavily granulated. Arrows point to the oocytes of appropriate quality. Scale 100 µm.

**Figure 4.**
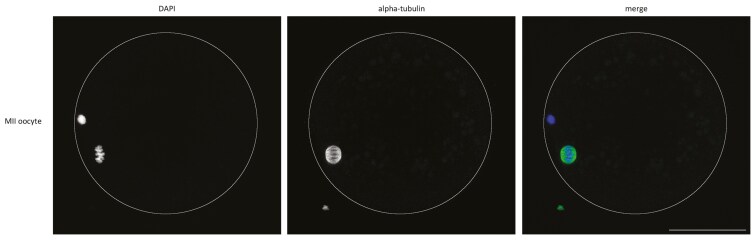
Representative images of immunofluorescence staining of alpha-tubulin in bovine MII oocytes 24 h after in vitro maturation. Blue = DNA (DAPI), green = alpha-tubulin. Scale bars indicate 50 µm.

The COCs were classified according to the appearance of their cumulus cells (Table 1 and Figure 1), and their expansion was evaluated (expanded/nonexpanded, Figure 2). Subsequently, denuded oocytes were also classified and divided into three categories based on the structure of their ooplasm (Table 2 and Figure 3), same as in Stadnik et al. (2024). The fertilization capability of the cow was defined as maturation of at least one aspirated oocyte to the MII stage (Figure 4).

**Table 1.** Classification of oocytes

	Class	Morphology of oocyte
Quality 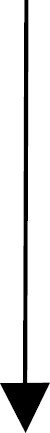	1	Compact, homogeneous, slightly granulated
	2	Granulated, heterogeneous
	3	Fragmented, heavily granulated

**Table 2.** Classification of COCs

Class	Number of cumulus layers
1	More than five layers
2	2 to 5 layers
3	One layer (also just partly surrounding)
4	No cumulus cells

### Immunofluorescent and lipid staining

Oocytes were fixed in 4% paraformaldehyde for 50 min at room temperature. After washing in phosphate-buffered saline, the oocytes were incubated in 0.5% (v/v) TritonX-100 for 15 min. All subsequent steps were done in PBS supplemented with 0.3% (w/v) BSA and 0.05% (w/v) saponin. Oocytes were blocked with 2% normal goat serum (Millipore Biosciences; St. Charles, MO) for 1 h and incubated with mouse antitubulin primary antibody (T6793; Sigma-Aldrich) overnight at 4 °C. After thorough washing, the oocytes were incubated with secondary antibody antimouse Alexa Fluor 488 (Invitrogen, Eugene, OR) for 1 hour at room temperature in the dark. For lipid visualization, oocytes were subsequently stained with Nile Red (Invitrogen) at a final concentration of 10 µg/mL in PBS supplemented with 0.4% BSA for 40 min, washed and mounted on glass slides using SlowFade Diamond Antifade Mountant with DAPI (Invitrogen). The samples were examined using Leica TCS SP5 (Leica Microsystems AG, Wetzlar, Germany). The images were processed using Image J software.

**Figure 5.**
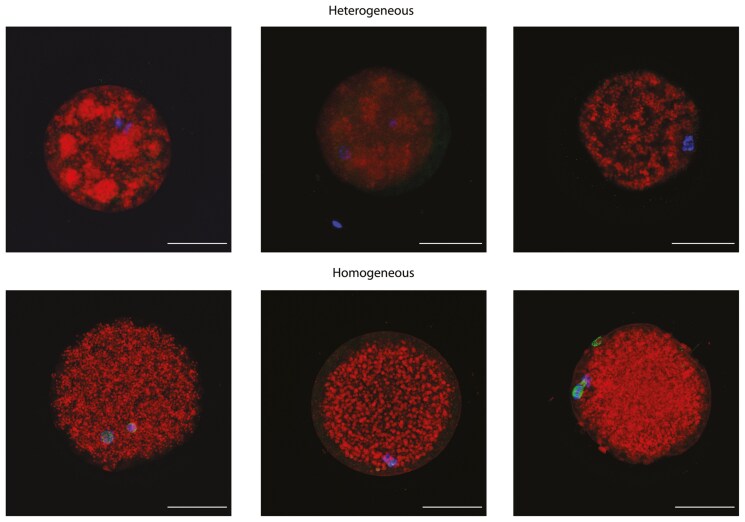
Representative images of lipid droplets distribution—heterogeneous and homogeneous. Blue = DNA (DAPI), red = lipid droplets (NileRed), green = alpha-tubulin. Scale bars indicate 50 µm.

The presence of a meiotic spindle and polar body was observed, the relative fluorescence of lipid content was measured, and lipid droplets were evaluated based on their localization (homogeneous and heterogeneous, Figure 5) and size (large, middle, and small).

### Statistical analysis

The SAS software ver. 9.4 (SAS/STAT; SAS Institute, Inc. Cary, NC, USA) was used for statistical analyses. The basic statistics were calculated by the UNIVARIATE procedure. Pearson correlation coefficients to measure the relationship between the analyzed variables were determined using the CORR procedure (number of small (<0.05 cm), large (>0.05 cm) and total follicles, number of days needed to restart the ovulation cycle, number of cycles needed to conceive, maturation rate, fat in milk, and fat to protein ratio (F/P) in milk gained at the day of aspiration, F/P 25 to 35 DIM, conductivity of milk, maximum, minimum, and mean level of F/P in milk 25 to 35 DIM). Multifactor analysis of variance was explored using the GLM procedure. The REG procedure, the STEPWISE method, and the Akaike information criterion parameter were used to select appropriate effects for the model equation. The groups of metabolites were divided according to arithmetic means and standard deviation (group 1: <−0.5 s; group 2: −0.5 s to +0.5 s; group 3: > +0.5 s). Differences between means were evaluated using the Tukey–Kramer test. The model equation for evaluation oocytes quality parameters was following:


$$\begin{array}{l} y_{ijkl}\;=\;\mu +MP_{i}+SE_{j}+OS_{k}+b*\left(DIM\right)+b*\left(ANIM\right)+e_{ijkl} \end{array}$$

where *y*_*ijkl*_—measured value of dependent variables (probability of reaching metaphase II; quality of oocytes; quality of COCs; expansion of cumulus cells; distribution of lipid droplets; relative fluorescent intensity of lipid staining;); *µ*—mean value of the dependent variable; MP*i*—fixed effect group of blood metabolite parameters (tChol concentration variant—*i* ≤ 3.927 mmol/L, *n* = 142; *i* = 3.927 to 4.903 mmol/L, *n* = 140; *i* ≥ 4.903 mmol/L, *n* = 86; TAGs variant—*i* ≤ 0.117 mmol/L, *n* = 131; *i* = 0.117 to 0.165 mmol/l, *n* = 122; *i* ≥ 0.165 mmol/L, *n* = 115; NEFA variant—*i* ≤ 0.399 mmol/L, *n* = 134; *i* = 0.399 to 0.743 mmol/L, *n* = 141; *i* ≥ 0.743 mmol/L, *n* = 93; BHB variant—*i* ≤ 0.238 mmol/L, *n* = 131; *i* = 0.238 to 0.350 mmol/L, *n* = 122; *i* ≥ 0.350 mmol/L, *n* = 108); SEj—fixed effect season of calendar year (*j* = 1—colder (October to March), *n* = 220; *j* = 2—warmer (April to September), *n* = 251); OSk—fixed effect oocyte maturation stadium (*k* = 0, *n* = 24; *k* = 1, *n* = 109; *k* = 2, *n* = 203; *k* = 3, *n* = 74); b*(DIM)—linear regression on DIM by oocyte aspiration; b*(ANIM)—linear regression on animals in repetition; *e*_*ijkl*_—random residual error.

The significance levels for all evaluations were set at *P* < 0.05 and *P* < 0.01.

## Results

### Descriptive statistics of milk and metabolic parameters related to oocyte yield

The average oocyte yield per aspirated primipary dairy cow was 5.35 with SD 4.6. The average TAGs, tChol, BHB, and NEFA content were 0.141 mmol/L, 4.415 mmol/L, 0.294 mmol/L, and 0.571 mmol/L in this order. The average F/P in milk gained 25 to 35 DIM was 1.07. The average milk yield until the first aspiration was 27.36 l. The average fat and protein in milk on the day of aspiration were in average 3.96 %, respectively 3.53%.

### The fertilization capability was higher during the warmer months of the year

To ascribe the seasoning effects on the reproduction ability, we divided the year into two seasons—warmer and colder. During the warmer months, the capability of the cow to be fertilized was higher (+0.1 to +0.16; *P* < 0.05), the distribution of lipid droplets was more often homogenous (+0.08 to +0.24; *P* < 0.001), and the intensity of lipid staining was lower (−5.3 to −8.8; *P* < 0.05).

### The fertilization capability was affected by the level of TAGs and BHB, not tChol and NEFA

To describe the metabolic profile of the lactating cows, the levels of several serum markers of lipid mobilization were characterized: namely tChol (mmol/L), TAGs (mmol/L), NEFA (mmol/L) and BHB (mmol/L).

The fertilization capability was higher in cows with higher levels of TAGs and BHB in blood (*P* < 0.05 in both cases), as can be seen in Table 3. The level of tChol and NEFA has no significant effect on the fertilization capability (*P* > 0.05).

**Table 3.** Impact of tChol, TAGs, NEFA, and BHB levels on quality and morphology of aspirated oocytes

	Capability of the cow to be fertilized^1^	Quality of oocytes^2^	Quality of COCs^3^	Expansion of cumulus cells^4^	Distribution of lipid droplets^5^	Relative fluorescent intensity of lipids (%)
**tChol**						
** Group 1** ≤3.927 mmol/L	0.76 ± 0.03	2.13 ± 0.08^a,b^	2.76 ± 0.11	0.26 ± 0.06^a^	1.33 ± 0.05^a,b^	64.46 ± 1.71
** Group 2** 3.927–4.903 mmol/L	0.81 ± 0.03	2,15 ± 0.08 ^b^	2,57 ± 0.11	0,40 ± 0.06^a,b^	1,37 ± 0.04^a^	61,13 ± 1.70
** Group 3** ≥4.903 mmol/L	0.75 ± 0.04	1.81 ± 0.09^c^	2.47 ± 0.14	0.48 ± 0.07^b^	1.22 ± 0.54^b^	66.83 ± 2.08
**TAGs**						
** Group 1** ≤0.117 mmol/L	0.74 ± 0.03^a^	2.12 ± 0.08	2.88 ± 0.11^A^	0.31 ± 0.07	1.35 ± 0.05	59.74 ± 1.76^A^
** Group 2** 0.117–0.165 mmol/L	0.73 ± 0.03^a^	1.96 ± 0.08	2.61 ± 0.11^A,B^	0.38 ± 0.06	1.24 ± 0.04	64.07 ± 1.66^A,B^
** Group 3** ≥0.165 mmol/L	0.88 ± 0.04^b^	2.09 ± 0.1	2.25 ± 0.14^B^	0.43 ± 0.07	1.39 ± 0.06	69.24 ± 2.12^B^
**NEFA**						
** Group 1** ≤0.399 mmol/L	0.82 ± 0.03	2.07 ± 0.08	2.63 ± 0.11	0.4 ± 0.06	1.27 ± 0.05	63.4 ± 1.72^a^
** Group 2** 0.399–0.743 mmol/L	0.76 ± 0.03	2.08 ± 0.07	2.64 ± 0.1	0.4 ± 0.06	1.37 ± 0.4	60.65 ± 1.58^A^
** Group 3** ≥0.743 mmol/L	0.74 ± 0.04	1.96 ± 0.09	2.54 ± 0.12	0.31 ± 0.07	1.28 ± 0.05	69.37 ± 1.88^B,b^
**BHB**						
** Group 1** ≤0.238 mmol/L	0.75 ± 0.03^a^	2.09 ± 0.08	2.63 ± 0.11	0.39 ± 0.06	1.3 ± 0.04	61.33 ± 1.64^a^
** Group 2** 0.238–0.350 mmol/L	0.74 ± 0.03^a^	1.2 ± 0.08	2.71 ± 0.12	0.34 ± 0.06	1.33 ± 0.05	63.85 ± 1.72^a,b^
** Group 3** ≥0.350 mmol/L	0.86 ± 0.03^b^	2.03 ± 0.08	2.47 ± 0.12	0.41 ± 0.64	1.29 ± 0.05	68.36 ± 1.77^b^

^1^Maturation of at least one aspirated oocyte to the MII stage.

^2^Explained in Table 1.

^3^Explained in Table 2.

^4^Expansion was evaluated as a binary parameter yes = 1/no = 0.

^5^Classified as homogenous (1) and heterogeneous (2).

^A,B^Different letters indicate statistical significance *P* < 0.01.

^a,b^Different letters indicate statistical significance *P* < 0.05.

Abbreviation: COCs, cumulus-oocyte complexes.

### The quality of oocytes and cumulus cells was affected by low levels of tChol and TAGs, respectively

The quality of oocytes and cumulus cells was always affected by only one of the studied metabolic markers: in the case of oocytes, lower levels of tChol were detected in cows with lower quality oocytes (*P* < 0.05); lower levels of TAGs were detected in cows with lower quality of cumulus cells (*P* < 0.001) (Table 4).

**Table 4.** Correlation between selected analyzed parameters

		Days until the estrus cycle after oocytes aspiration	Number of estrus cycles to get pregnant after oocytes aspiration	Number of aspirated oocytes per cow	Fertilization capability	Probability of reaching metaphase II (%)	Quality of oocytes	Quality of COCs	Expansion of cumulus cells	Relative fluorescent intensity of lipids (%)	Distribution of lipid droplets
tChol	r	0.004	0.260	−0.103	−0.146	−0.202	−0.024	−0.003	0.088	0.145	0.054
	P	0.948	<0.001	0.050	0.007	<0.001	0.657	0.951	0.153	0.008	0.319
	n	343	273	365	344	346	348	348	262	340	338
TAGs	r	0.116	0.069	0.324	0.148	−0.119	−0.099	−0.238	0.083	0.124	−0.084
	P	0.032	0.258	<0.001	0.006	0.027	0.064	<0.001	0.178	0.022	0.125
	n	343	273	365	344	346	348	348	262	340	338
NEFA	r	−0.082	−0.017	−0.078	−0.032	−0.034	0.006	−0.034	−0.060	0.191	0.100
	P	0.132	0.782	0.139	0.559	0.526	0.908	0.531	0.332	<0.001	0.067
	n	343	273	365	344	346	348	348	262	340	338
BHB	r	0.014	0.409	0.139	0.065	−0.017	−0.010	−0.055	−0.010	0.195	0.057
	P	0.792	<0.001	0.008	0.230	0.748	0.851	0.304	0.877	<0.001	0.299
	n	343	273	365	344	346	348	348	262	340	338

Abbreviations: tChol, total cholesterol (mmol/l); TAGs, triacylglycerols (mmol/l); NEFA, nonesterified fatty acids (mmol/l); BHB, beta-hydroxybutyrate (mmol/l); r, correlation coefficient; *P*, statistical significance; n, number of observations.

### High levels of TAGs and tChol in serum correlated with low maturation yields

The high levels of TAGs correlated with a lower maturation rate (*P* < 0.05) although the high levels also correlated with a higher quality of cumulus cells surrounding the aspirated oocyte (*P* < 0.001), which is typical for oocytes with higher maturation probability. Moreover, high TAG levels correlated with a higher number of aspirated oocytes per cow (*r* = 0.324; *P* < 0.001) and with higher fertilization capability (*r* = 0.148; *P* < 0.01).

Similar negative correlation trend with oocyte maturation as in comparison to TAGs levels was found in comparison to tChol levels (*P* < 0.05), but a lower number of oocytes was aspirated (*P* < 0.05), even though the quality of the aspirated oocytes was higher and the cumulus cells expanded in a larger number COCs (*P* < 0.05) (Table 4).

There was no statistically significant correlation between oocyte maturation rates vs. BHB and NEFA levels (*P* > 0.05 in each case); however, high BHB levels correlated with a higher number of aspirated oocytes (*P* < 0.05).

### The levels of metabolic profile markers affected the relative fluorescence of lipid staining signal but not lipid-droplet distribution

Further, we wanted to see whether the content of lipid droplets in the oocyte was connected to the level of metabolic profile markers tChol, TAGs, BHB, and NEFA in blood. In cows with higher levels of TAGs and BHB (group 1 vs. 3) and NEFA (group 2 vs. 3) in blood, the intensity of lipid staining in the oocytes was higher (*P* < 0.05 in each case) (Table 3). The high lipid levels in oocyte also correlated with the quality of cumulus cells (*P* < 0.05) and gained oocytes (*P* < 0.05). On the other hand, the level of neither tChol, TAGs, BHB, nor NEFA affected the distribution of lipid droplets in the oocyte (*P* > 0.05 in each case). This was supported by the correlation in Table 4.

### Higher levels of TAGs and tChol were present in cows with a higher amount of small follicles

Further, we wanted to see the impact of the levels of lipid mobilization markers (tChol, TAGs, NEFA, and BHB) on follicle growth. Most prominent was the correlation of the number of small follicles (<0.05 cm) and total tChol (*P* < 0.05) and TAGs (*P* < 0.001) levels (Table 5). Similar correlation was found in total number of follicles vs. the level of tChol and TAGs (*P* < 0.05), which is likely a consequence of the increase in the number of small follicles. Neither the number of all follicles nor of small follicles correlated with the BHB or NEFA level (*P* > 0.05 in each case). On the other hand, the number of large follicles (>0.5 cm) significantly correlated only with NEFA levels (*P* < 0.05).

**Table 5.** Correlation between selected analyzed parameters

		Number of follicles	Number of small follicles (<0.5 cm)	Number of large follicles (>0.5 cm)	Milk yield (kg) on the day of aspiration	F/P in milk 25–35 DIM	F/P in milk before first aspiration	Fat (%) on the day of aspiration	Protein (%) collected on the day of aspiration
tChol	r	0.125	0.109	0.014	0.291	−0.118	−0.193	−0.210	0.016
	P	0.016	0.036	0.791	<0.001	0.023	<0.001	<0.001	0.756
	n	368	368	368	366	368	366	366	366
TAGs	r	0.170	0.177	−0.083	−0.060	−0.159	0.180	0.106	−0.200
	P	0.001	0.001	0.113	0.249	0.002	0.001	0.042	0.000
	n	368	368	368	366	368	366	366	366
NEFA	r	0.041	−0.001	0.129	0.101	−0.011	0.057	0.010	−0.100
	P	0.432	0.992	0.013	0.054	0.838	0.278	0.842	0.055
	n	368	368	368	366	368	366	366	366
BHB	r	−0.051	−0.019	−0.092	0.140	−0.037	0.192	0.143	−0.186
	P	0.331	0.719	0.077	0.007	0.479	0.000	0.006	0.000
	n	368	368	368	366	368	366	366	366

Abbreviations: tChol, total cholesterol (mmol/l); TAGs, triacylglycerols (mmol/l); NEFA, nonesterified fatty acids (mmol/l); BHB, beta-hydroxybutyrate (mmol/l); r, correlation coefficient; *P*, statistical significance; n, number of observations; F/P, fat to protein ratio in milk.

### The levels of selected metabolic marker affected the cycle, lactation, and the capability to conceive

As we have found that the level of metabolic markers affects maturation, fertilization, and quality of oocytes and cumulus cells, we wanted to know whether metabolic markers also affect the reproduction ability of the cow. In cows with high levels of TAGS in blood, a higher number of days was needed to restart the ovulation cycle (*r* = 0.12; *P* < 0.05). High levels of tChol and BHB correlates (*r* = 0.26; resp. *r* = 0.41) with the number of cycles needed to conceive (*P* < 0.001) (Table 4). No statistically significant correlation with reproduction ability markers was found in other metabolic markers (*P* > 0.05 in each case).

### The level of tChol in blood negatively correlated with level of fat in milk

We further wanted to know whether the levels of lipid-metabolic markers in blood are related not only to reproductive competence of the cow but also to quality of gained milk represented by fat levels in milk. Interestingly, the levels of tChol negatively correlated with levels of fat in milk collected on the day of aspiration (both fat and F/P ratio) and F/P in milk gained 25 to 35 DIM (*P* < 0.05 in each case) (Table 5). On the other hand, the levels of TAGs and BHB correlated with the levels of fat and F/P ratio in milk on the day of aspiration and conductivity on the day of aspiration (*P* < 0.001) but negatively correlated with the maximum level of F/P ratio in milk gained before the first aspiration (*P* < 0.001 in each case). The TAGs levels also negatively correlated with the mean F/P in milk gained before first aspiration and F/P ratio in milk gained 25 to 35 DIM (*P* < 0.001 and *P* < 0.05, respectively) (Table 5).

## Discussion

The aim of this study was to find the relation between the profile of lipid-metabolic markers (tChol, TAGs, BHB, and NEFA) of the lactating primiparous dairy cows and the oocyte maturation and fertilization potential of oocytes obtained via OPU 36 to 75 DIM and matured in vitro. The reproduction serum markers affecting the highest number of processes were shown to be the tChol and the TAG level. Both indicators correlate with low maturation rates but while high TAGs levels seem to have positive effect in most other reproductive processes, high tChol levels seem to deteriorate the reproductive capacity of cows as it lowers the number of aspirated oocytes and increases the number of cycles needed to conceive, even though the quality of oocytes is higher. High TAG level, on the other hand, correlates with high fertilization capability, quality of cumulus cells, number of aspirated oocytes and intensity of lipid staining. Moreover, the lower maturation rate, which shows the percentage of oocytes reaching the MII stage from all aspirated oocytes, is not necessarily a negative marker as a singleton pregnancy is desired. Thus, it seems that TAG levels can be used as a marker of reproduction capability of the cow.

Bovine oocytes are among the richest in lipid in the mammalian kingdom, with TAGs known as the most abundant. The content of lipids in oocyte slightly increases during oocyte maturation (Aardema et al., 2011) and during in vitro fertilization, the content of lipids is highly affected by the maturation environment (reviewed in Nemcova et al., 2023). Murine oocytes are supplied with cholesterol, and probably other lipids, by cumulus cells (Su et al., 2008; Dunning et al., 2014). This is likely how the quality of cumulus cells is related to the lipid staining intensity of oocytes. The level of at least TAG and NEFA levels correlates in serum and follicular fluid in dairy cattle (Leroy et al., 2004; Jungheim et al., 2011; Valckx et al., 2012), which explains the impact of lipid-metabolic markers on maturation and conception success. The lipids in oocyte serve as a source of energy for oocyte and preimplantation embryo (Su et al., 2008; Dunning et al., 2014). Even though we have found a clear correlation between intensity of lipid staining and oocyte quality, the lipid content does not seem to be the main biomarker of the oocyte/embryo quality and high accumulation of lipid droplets in bovine oocytes results in lower cryotolerance and marks thus lower ability of the oocyte to be fertilized (Dunning et al., 2014). Interestingly, the levels of lipid-metabolism markers in blood have no impact on the distribution of lipid droplets in oocyte.

The level of BHB and NEFA had an impact on less processes connected to reproductive ability of the cow. Nevertheless, NEFA is thought to support the follicle maturation (Renaville et al., 2010), which is supported by our finding that high NEFA levels in serum correlated with higher number of large follicles. Thus, NEFA does play an important role in the determination of the reproduction capability as well. The high BHB levels were found in cows with high fertilization capability but also increased the number of cycles needed to conceive and rather than reproduction, BHB affects the fat content of the milk.

After in vitro maturation of oocytes with higher NEFA level, blastocysts derived from these oocytes showed a lower cell number, increased apoptotic cell index, signs of glucose intolerance, sensitivity to oxidative stress, and mitochondrial dysfunction (Leroy et al. 2018). According to the results of De Bie et al. (2017) elevated NEFA concentrations disrupt oocyte and embryo development and quality. In vitro maturation with an increased concentration of BHB in the medium did not affect the rate of oocyte maturation, but there was a concentration-dependent decrease in the ability of fertilized oocytes that developed to the blastocyst stage (Sarentonglaga et al., 2013). An in vivo study by Matoba et al. (2012) investigated the relationship between metabolic parameters (levels of NEFA, BHB, insulin, insulin-like growth factor 1, and glucose) and the quality of oocytes collected by aspiration. A major finding of this study is that despite the increase in NEFA, BHB, and other metabolic changes associated with calving and the peak of lactation in the weeks after parturition, oocyte and embryo quality, as assessed morphologically in terms of fertilization ability and development, was not affected up to the blastocyst stage.

The results show that the season significantly affects selected oocyte aspiration parameters, which may be related to heat stress. The effect of heat stress on production was confirmed by e.g., Chen et al. (2024) and for the reproduction Bezdíček et al. (2024).

On the day of aspiration, milk with higher fat content and conductivity was gained in cows with higher BHB and TAG levels. On the other hand, this was not true for the F/P ratio of milk before the first aspiration, where the trend was rather the opposite. In cows with higher tChol levels, the fat content in milk and conductivity of the milk were lower. As the higher F/P ratio correlates with the number of aspirated oocytes (Stádník et al., 2024) this confirms BHB and TAG levels as useful marker of reproduction capability. NEFA serum levels had no effect on fat levels in milk.

In conclusion, the reproduction potential of the primiparous dairy cows was affected by the season in which the aspiration was performed. Better results of fertilization were achieved during warmer months, and this was further supported by the distribution of lipids in oocyte during this period (homogenous distribution in warm months). Various correlations between metabolic indicators and oocyte maturation parameters were also confirmed. In conclusion, the high levels of lipid-metabolic markers such as TAGs, BHB, and NEFA can be used as positive markers of the readiness of the cow to become pregnant. However, the impact of tChol is ambiguous and thus cannot be used as reproductive marker. This provides a prerequisite for using this information to improve the reproduction efficiency of dairy cattle.

## Abbreviations

BHB: beta-hydroxybutyrate
COC: cumulus-oocyte-complex
DAPI: 4′,6-diamidin-2-fenylindol
DIM: days in milk
F/P: fat to protein ration
GLM: generalized linear model
GV: germinal vesicle
MII: metaphase of second meiotic division
NEB: negative energy balance
NEFA: nonesterified fatty acid
OPU: ovum pick-up
TAG: triacylglycerol
tChol: total cholesterol
